# An Update of the Virion Proteome of Kaposi Sarcoma-Associated Herpesvirus

**DOI:** 10.3390/v12121382

**Published:** 2020-12-02

**Authors:** Ramina Nabiee, Basir Syed, Jesus Ramirez Castano, Rukhsana Lalani, Jennifer E. Totonchy

**Affiliations:** Biomedical and Pharmaceutical Sciences Department, Chapman University School of Pharmacy, Irvine, CA 92618, USA; nabie100@mail.chapman.edu (R.N.); bsyed@chapman.edu (B.S.); jcastano@chapman.edu (J.R.C.); rukhsana_lalani@yahoo.com (R.L.)

**Keywords:** KSHV virion proteome, viral proteomics, bottom-up shotgun proteomics, UHR-QqTOF

## Abstract

The virion proteins of Kaposi sarcoma-associated herpesvirus (KSHV) were initially characterized in 2005 in two separate studies that combined the detection of 24 viral proteins and a few cellular components via LC-MS/MS or MALDI-TOF. Despite considerable advances in the sensitivity and specificity of mass spectrometry instrumentation in recent years, leading to significantly higher yields in detections, the KSHV virion proteome has not been revisited. In this study, we have re-examined the protein composition of purified KSHV virions via ultra-high resolution Qq time-of-flight mass spectrometry (UHR-QqTOF). Our results confirm the detection of all previously reported virion proteins, in addition to 17 other viral proteins, some of which have been characterized as virion-associated using other methods, and 10 novel proteins identified as virion-associated for the first time in this study. These results add KSHV ORF9, ORF23, ORF35, ORF48, ORF58, ORF72/vCyclin, K3, K9/vIRF1, K10/vIRF4, and K10.5/vIRF3 to the list of KSHV proteins that can be incorporated into virions. The addition of these proteins to the KSHV virion proteome provides novel and important insight into early events in KSHV infection mediated by virion-associated proteins. Data are available via ProteomeXchange with identifier PXD022626.

## 1. Introduction

Kaposi’s sarcoma herpes virus (KSHV) was first discovered about twenty-five years ago through the use of representational difference analysis by Chang et al. [1], and about a year later, Moore et al. [2] looked into similarities of this novel virus with other known gammaherpesviruses and were able to identify it as the most similar with herpesvirus saimiri (HVS) through gene alignment and amino acid sequencing alignments on Open Reading Frames (ORFs) suitable for phylogenetic analysis; however, they noted that the divergence between the viruses is ancient. They, furthermore, classified this virus as the first human gamma-2 herpesvirus. To this day, KSHV is the only viral agent of the rhadinoviral subfamily capable of infecting human B cells [1]. In the context of immunosuppression, KSHV infection causes Kaposi’s sarcoma, an endothelial cell neoplasm, the B cell lymphoproliferative disorders primary effusion lymphoma (PEL) and multicentric Castleman’s disease (MCD), as well as a recently discovered KSHV inflammatory cytokine syndrome disease (KICS) [3].

As with all herpesviruses, KSHV undergoes either latent or lytic infection programs. New virions are produced during the lytic phase, and latency is used for long-term maintenance of viral genomes as extra-chromosomal episomes [4]. Although different environmental and physiological signals, such as viral-coinfections [5], hypoxia [6], oxidative stress [7]; cellular factors and cellular signaling [8], such as histone deacetylases [9], and even dietary supplements [10] can cause KSHV lytic reactivation, latency is thought to be the predominant mode of KSHV infection in human hosts. However, there is convincing evidence that lytic replication plays a significant role in KSHV-associated pathology [11]. Despite the substantial body of work on KSHV, early infection events in disease-relevant cell types and the role of individual virion-associated tegument factors in the establishment of infection remain poorly studied [12].

The tegument layer is a dense proteinaceous layer sandwiched between the nucleocapsid and the virion envelope that can critically affect the host’s cellular and viral biology immediately following viral entry [13]. These proteins make up a large proportion of the virion’s overall protein composition, and they serve as prepackaged factors capable of influencing the host cell physiology immediately upon the fusion of the viral envelope with the host cell membrane. Early infection functions of tegument proteins include cell cycle modulation [14], transcriptional activation of viral genes [15], inhibition of host gene expression activation [16], translocation of the virion capsid to the nucleus [17], and evasion of the innate immune response. Thus, identification and functional analysis of the virion proteins of KSHV is a critical step in understanding the early stages of KSHV infection.

In 2005, two research groups independently characterized the virion proteins of KSHV derived from lytic induction of latently infected BCBL-1 PEL cells. These studies both utilized a bottom-up proteomics approach to characterize virion-associated proteins by extracting individual protein bands visible on SDS-PAGE gels of purified virions, using either MALDI-TOF [18] or LC, MS/MS [19] to identify the individual proteins after tryptic digestion. These studies together identified 24 viral and a few cellular proteins as virion associated. Additional proteins have since been detected in KSHV virions via other methods [20,21,22,23,24,25,26,27]; however, despite significant advances in proteomics methods and instrumentation in recent years, the KSHV virion proteome has not been revisited.

Advances in proteomics technology have given rise to de novo sequencing in shotgun proteomics [28], which combines high-resolution spectrum data from modern mass spectrometry instruments with automated de novo peptide sequencing algorithms. These technological advances, combined with enhancements in sample preparation, have revolutionized proteomics in the last ten years.

In this study, we have taken advantage of advanced instrumentation and analysis methods; to reanalyze the KSHV virion proteome using a bottom-up shotgun method [29], combined with de novo sequencing on highly purified KSHV virions produced from the iSLK the epithelial cell line as well as dual digestion with trypsin/lys-c and chymotrypsin. Our method’s improved sensitivity has allowed us to validate all of the 24 previously reported virion-associated proteins and add seventeen other proteins to the KSHV virion proteome. Seven of our novel proteomic hits have been identified as virion-associated by non-proteomic methods, and we report ten proteins in our analysis that have never previously been identified as virion-associated.

## 2. Materials and Methods 

### 2.1. Purification of Cell-Free KSHV Virions

Latently infected iSLK producer cells stably infected with BAC16 recombinant KSHV WT were grown to 70–75% confluency in DMEM (Caisson Labs, Smithfield, UT, United States Catalog#: DML10—500 ML) supplemented with 10% cosmic calf serum (Sigma-Aldrich, St. Louis, MO, United States Catalog#: C8056—500 ML), 250 µg/mL geneticin (Goldbio, St. Louis, MO, United States Catalog#: G-418-1), 1 µM puromycin (Bio Basic, Markham, ON, Canada Catalog#: PJ593), and 1.2 mg/mL hygromycin B (A.G. Scientific, San Diego, CA, United States Catalog#: H-1012-PBS). Then induced for 72 h with 3 mM sodium butyrate (Sigma-Aldrich, St. Louis, MO, United States Catalog#: B5887-1G) and 2 µM doxycycline (Tocris, Minneapolis, MN, United States Catalog#: 4090—50 mg). Culture supernatants were clarified by centrifugation at 500 g for 12 min and passed through a 0.45 uM filter placed on ice. The virus was then pelleted out of clarified supernatants over a 50% Opti-prep (Iodixanol) [30] cushion prepared in TNE buffer at pH 7.40 by ultracentrifugation at 41,000 g for 120 min using an SW28 rotor. Immediately after (adapting a previously reported purification method used for mass spectrometry of virions), the concentrated virus particles were centrifuged through a 10–50% Iodixanol density step gradient prepared in TNE buffer [31] at 45,000 g for 2 h. The virus band at the gradient junctions of 22–24% was collected and kept at −80 °C for further processing. Iodixanol fractions were also collected and the viral DNA was extracted from them using the Zymo Quick DNA/RNA Viral kit (Zymo Research, Irvine, CA, United States Catalog#: D7020). Endpoint RFU qPCR assay was then performed on DNA fractions using probes and primers against LANA (LANA forward (5′-AATGGGAGCCACCGGTAAAG-3′), LANA reverse (5′- CGCCCTTAACGAGAGGAAGT-3′), LANA probe (5′ [6FAM]-ACACAAATGCTGGCAGCCCG-[BHQ1]3′)) using TaqProbe 5× qPCR MasterMix-Multiplex (ABM MasterMix-5PM), 5% DMSO, primers at 900 nM and probes at 250 nM on BioRad(Hercules, CA, United States ) CFX qPCR machine in 40 cycles.

### 2.2. Trypsin and Detergent Treatment of Purified Virions

The purified virion sample was then divided into two fractions. One fraction was treated with 0.25% trypsin- 2.21 mM EDTA for 60 min at 37 °C, while shaking at 300 rpm, to cleave and strip external envelope proteins and non-viral proteins and debris from virion preparations followed by virion lysis. This fraction is herein referred to as VTT fraction. The second fraction was kept untreated and is herein referred to as VT fraction. Both fractions were then treated with Pierce protease inhibitor (Thermo Scientific, Waltham, MA, United States Catalog#: A32963). Proteins were extracted and lipids removed using ReadyPrep 2D-Cleanup Kit (BioRad, Hercules, CA, United States Catalog#: 1632130). Protein concentration was then quantified using a Qubit 3.0 fluorometer (Invitrogen, Waltham, MA, United States). The sample concentration was adjusted to a minimum of 0.5 mg/mL and pH as adjusted to (pH 7.5). The purified proteome samples were reduced and alkylated, using Thermofisher EasyPep™ Mini MS Sample Prep Kit (Thermo Fisher Scientific, Waltham, MA, United States Catalog#: A40006).

The samples were then either tryptic digested for 6–8 h at 37 °C in Tris-HCl buffer with final protease to a protein ratio of 1:100 w/w of Pierce Trypsin/Lys-C to protease mix (Thermo Scientific, Waltham, MA, United States Catalog#:1863467) or digested with Pierce Chymotrypsin Protease (TLCK-treated) (Thermo Fisher Scientific, Waltham, MA, United States Catalog#: 90056) with final protease to protein ratio of 1:100 w/w, for 12–14 h at 37 °C in 1 mM HCl, while shaking at 300 rpm. The digestion was stopped using the digestion stop reagent of the EasyPep™ Mini MS Sample Prep Kit, and the samples were further cleaned and filtered following the kit’s protocol. The samples were then extracted in 0.1% formic acid in diH2O and kept at −80°C until analysis.

### 2.3. Mass Spectrometry Analysis

The samples were thawed on ice, and 50 uL of the peptide digest fraction was then injected onto a reverse-phase C18 Acclaim RSLC 120 column (Thermo Scientific, Waltham, MA, United States Catalog#:074812) at 2.2 um and 120°A 2.1 × 250 mm connected to an ultra-high performance liquid chromatography (UHPLC) coupled to an electrospray ionization source of an ion trap mass spectrometer (Bruker Impact II UHR-QqTOF LC/MS). The sample was separated within 75 min with 0.2 mL/minute flow rate. A gradient method started at 99% buffer A (0.1% Formic acid in water) and 1% buffer B (0.1% Formic acid in Acetonitrile) and held at 1% B for 5 min. The gradient was changed linearly to 55% B in 60 min, and then changed to 90% B in 10 min. Finally, it was held for 5 min to equilibrate back to 1% B initial condition. The column oven set at 37 °C.

QTOF parameters for electrospray capillary at 4.5 V dry gas was set to 8 L/min. Dry temperature was set to 200 °C. Nebulizer was set to 1.8 Bar. Target mass range was set from 300 to 1600 m/z. Collision cell energy was set to 8 eV. Transfer time was set to 90 µs, and pre-pulse storage was set to 10 µs. Spectra rate was set to 2 Hz with Auto MS/MS Spectra rate upper limit of 30 and lower limit of 4 and only the most intense precursor ions were selected for fragmentation and cycle time of 3 s. Dynamic exclusion duration was 0.3 min and exclude singly charged m/z. Precursor ions isolation was m/z dependent window of 2–5. The collision energy was adjusted, as a function of m/z value, between 23–65 eV, the system was calibrated using 10 mM sodium formate solution, and the accepted range was set to 250–1600 m/z.

Utilizing PEAKS DB, PEAKS PTM, and SPIDER search tools from PEAKS Studio (software version10)(78), the mzxml files were analyzed against a sequence database with the HHV8 protein database’s customized library pulled from Uniprot (Swiss-prot) (https://www.uniprot.org/uniprot/?query=reviewed:yes%20taxonomy:37296#) downloaded on 07/01/2020 containing 86 sequences. Trypsin (for trypsin digested samples) and chymotrypsin (for samples digested with chymotrypsin) were selected with a maximum of three missed cleavages permitted; the search parameters were set to fixed modification of Carbamidomethyl and variable modification of methionine oxidation. Additional criteria used in the search were peptide mass tolerance of ±20.0 ppm, and fragment mass tolerance of ±0.05 Da followed with the peptide score *p*-value of less than 0.00316. Using the Spider search feature, a special feature in PEAKS software, a single amino acid mutation and up to three additional modifications per peptide were automatically detected with PTM_Ascore of 20 and mutation ion intensity of 5%. All of reported peptides (Tables S1–S6) have a score (−10lgP) greater than 25 and corresponding to a statistically significant (*p* < 0.00316) confident identification; moreover, from our positive matches, only proteins identified with more than at least 3 peptide sequences with a mass tolerance of <0.05 Da were reported. An exception was made for the two previously reported ORF53 and ORF28, which are very small in size and contain a limited number of trypsin cleavage sites when using trypsin. However, using chymotrypsin, we were able to report more hits for both proteins. Additionally, we queried a common contaminants database to ensure the overall quality of the samples. The mass spectrometry proteomics data have been deposited to the ProteomeXchange Consortium via the PRIDE [32] partner repository with the dataset identifier PXD022626 and 10.6019/PXD022626 [33,34].

### 2.4. Antibodies and Western Blotting

Purified virion proteome equivalent to 1:50 of the final yield was mixed 1:1 with 5× Laemmeli buffer (0.5M Tris-HCl PH6.8, Glycerin, S.D.S., 0.25%Bromophenol blue, B-mercaptoethanol) boiled for 10 min at 95 °C and then run on Mini-Protean T.G.X. Stain Free 4–15% Bis-Tris PAGE gels (BioRad, Hercules, CA, United States, Catalog#:4561081), then transferred to chemiluminescence Polyvinylidene difluoride(PVDF) membranes (Amersham, Buckinghamshire, United British Kingdom, Catalog#:88585). The membranes were dry-blocked overnight and rehydrated with 2% dried milk in T.B.S. buffer and then incubated with diluted primary antibodies against ORF8 (gB) (Invitrogen, Waltham, MA, United States, Catalog#:PA5-19852) or B-actin (Cell Signaling technology, Danvers, MA, United States, Catalog#8H10D10) as the housekeeping control for four h at room temperature or 4 °C overnight. Anti-rabbit or anti-mouse immunoglobulin G antibody conjugated to horseradish peroxidase (Invitrogen, Waltham, MA, United States) was used as the secondary antibody. The enhanced chemiluminescence system (BioRad, Hercules, CA, United States, Catalog#: 1705060S) was used to detect antibody–antigen complexes using the BioRad Universal Hood II gel imager.

For the iodixanol fractions each fraction was collected then mixed 1:1 with 5x Laemmeli buffer (0.5M Tris-HCl PH6.8, Glycerin, S.D.S., 0.25% Bromophenol blue, B-mercaptoethanol) boiled for 10 min at 95 °C and then run on two similar Mini-Protean T.G.X. Stain Free 4–15% Bis-Tris PAGE gels (BioRad, Hercules, CA, United States, Catalog#:4561081), post run one of the gels were stained with Coomassie blue (0.05% Coomassie brilliant blue, 50% methanol, 10% acetic acid) for 1 h, and destained (50% methanol, 10% acetic acid) for 3 h. The second gel was then transferred to chemiluminescence PVDF membranes (Amersham, Buckinghamshire, United British Kingdom, Catalog#:88585). The membrane was dry-blocked overnight and rehydrated with 2% dried milk in T.B.S. buffer and then incubated with diluted primary antibody against ORF8 (gB) (Invitrogen, Waltham, MA, United States, Catalog#:PA5-19852) four hours at room temperature or 4 °C overnight. Anti-rabbit G antibody conjugated to horseradish peroxidase (Invitrogen, Waltham, MA, United States) was used as the secondary antibody. The enhanced chemiluminescence system (BioRad, Hercules, CA, United States, Catalog#: 1705060S) was used to detect antibody–antigen complexes using C-DiGit Blot Scanner (Li Cor Biosciences, Lincoln, NE, United States).

## 3. Results

### 3.1. Purification of Cell Free KSHV Virions and Virion Protein Fractions

**Figure 1:**
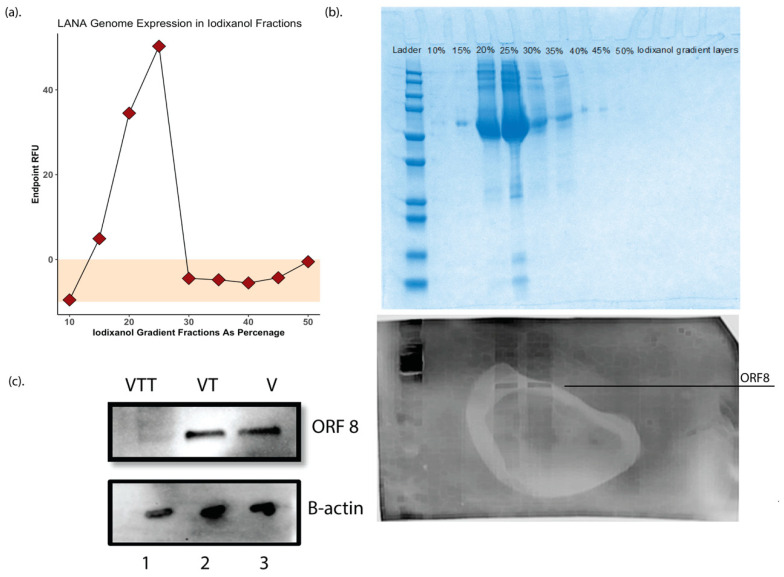
(**a**) Endpoint RFU qPCR using LANA genome on Iodixanol fractions. (**b**) Top Coomassie blue staining of the Iodixanol gradient fractions. (**b**) Bottom Western blot using gB/ORF8 antibody on different iodixanol gradient fractions to ensure the quality of the virion purification, based on the data, only fractions of 22–24% Iodixanol containing virions was used for further processing. This fraction was labeled as fraction V. (**c**) Western blot for gB/ORF8 on proteins from trypsin digested (VTT) and purified (VT) fractions as well as virions.

One of the significant limitations of the original KSHV proteomics studies was the production of high-yield purified virions from BCBL1 cells sufficient to obtain a high protein concentration for analysis. In this study, we employed the more recent and highly productive iSLK producer cell line to produce KSHV virions from the recombinant BAC16 KSHV genome for our studies [35]. iSLK cells were induced at confluency with doxycycline and sodium butyrate, and at 72 h post-induction, virions were purified from 2 L of filtered culture supernatants for further processing. Adopting a method previously reported for KSHV virion purification for mass spectrometry [31], the supernatant was then ultracentrifuged over a 50% Iodixanol (Opti prep) [30] cushion, the cell free virions in 50% iodixanol were collected and further purified by ultracentrifugation on a 10–50% Opti-Prep in TNE buffer density step gradient to ensure further cleanup of the sample. Virus was quantitated by real-time qPCR using LANA as a genomic target. A genome peak was obtained at the 22–24% Opti-Prep interface (Figure 1a). Fractions were then used in Coomassie blue staining and Western blotting against the ORF8(gB) antibody to determine the purity and concentration of virions in these fractions (similar to methodology used in Bechtel et al. [18]), respectively, (Figure 1b), and only the fractions around 22–24% iodixanol were collected and labeled as V fraction and kept at −80 °C for further processing.

The V fraction was then divided into two equal fractions, one where the proteins were extracted and then digested with either trypsin/LysC or chymotrypsin, referred to as VT fraction. The second was exposed to 0.25% trypsin- 2.21 mM EDTA for 60 min at 37 °C, while shaking at 300 rpm to strip the envelope layer and possible cellular debris before protein extraction and purification using labeled as the VTT fraction. The quality and purity of these fractions and the efficacy of external trypsin treatment was validated by Western blotting for ORF8/gB and B-actin (Figure 1c).

### 3.2. Mass Spectrometry Quality Control

As the VT fraction contains both glycoproteins as well as envelope contained proteins, it was used for the initial mass spectrometry analysis of probing for previously reported virion proteins. PEAKS Studio software (Version 10) [36] was used for all of the data analysis presented herein. Using the database search approach, the raw datafiles were analyzed against a customized library of HHV8 protein database pulled from Uniprot (Swissprot) ((https://www.uniprot.org/uniprot/?query=reviewed:yes%20taxonomy:37296#) downloaded on 07/01/2020 containing 86 sequences). Trypsin (for trypsin digested samples) and chymotrypsin (for samples digested with chymotrypsin) were selected with a maximum of three missed cleavages permitted; the search parameters were set to fixed modification of carbamidomethyl and variable modification of methionine oxidation. Additional criteria used in the search were peptide mass tolerance of ±20.0 ppm, and fragment mass tolerance of ±0.05 Da followed with the peptide score *p*-value of less than 0.00316. Using the Spider search feature, a special feature in PEAKS software, a single amino acid mutation and up to three additional modifications per peptide were automatically detected with PTM, a score of above 20 and mutation ion intensity of 5%. All of the reported peptides (included in Tables S1–S6) have a score (−10logP) greater than 25, corresponding to a statistically significant (*p* < 0.00316) confident identification. Moreover, from our positive matches, only proteins identified with more than at least three peptide sequences with a peptide mass tolerance of <20 ppm detected in triplicate runs were reported. It should be noted that similar to Zhu et al. [19], for the previously reported ORFs 28 and 53, due to the smaller size of the proteins, and limited trypsin cleavage cites, an exception was made were two and one peptide hits with trypsin were also accepted; however, using chymotrypsin, we detected three and two unique peptide hits, respectively.

**Figure 2:**
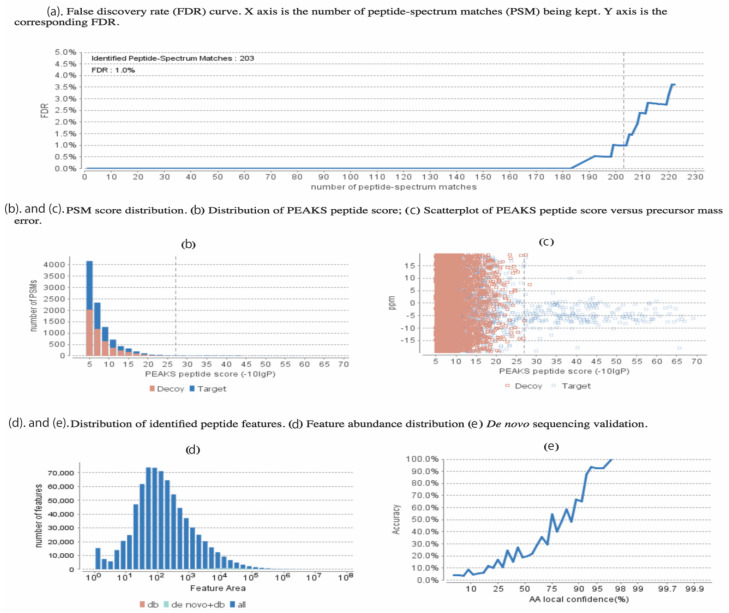
Statistical data presented from PEAKS analysis on the 17 proteins found uniquely in our study, with false discovery rate (FDR) set at 1%. (**a**) The false discovery rate (FDR) curve. The *X*-axis is the number of peptides-spectrum matches (PSM) being kept. The *Y*-axis is the corresponding FDR for the unique hits of our study. (**b**) Peptide-spectrum matches (PSM) score distribution, showing the distribution of the PEAKS peptide score. (**c**) The precursor mass error in ppm vs. −10lgP peptide score for all the PSMs. (**d**) Distribution of abundance of identified peptides. (**e**) The de novo sequencing validation and accuracy level (ALC).

An example of the quality control statistics and our applied methodology is shown in Figure 2. At an false discovery rate (FDR) = 1.0% (Figure 2a), 203 peptide matches were accepted following the other criteria including the –log p, which is set at 25 (Figure 2b) corresponding to the *p*-value of less than 0.01, demonstrating our sacrifice in reduction in the decoy hits in exchange for increase in true peptide match score. This decrease of *p*-value indicating the ppm peptide error is demonstrated and the blue dots are the high-quality hits (Figure 2c). Although the PEAKS DB database search feature was mainly used for our analysis, the PEAKS de novo sequencing feature was also used and Table S8 shows a few examples of some of our sequences with the ALC% cut-off of 75%. Figure 2d,e respectively show the distribution of our detected peptide features as well as the increasing trend in the accuracy of the de novo peptide match sequencing with a tight error tolerance.

### 3.3. KSHV Virion Protein Identification

**Figure 3:**
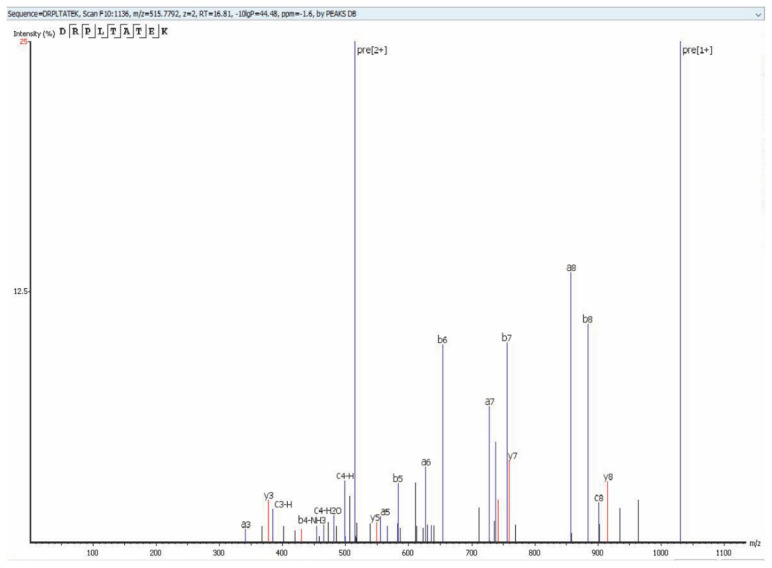
CID spectrum of the precursor ion at m/z7792 (z = 2) corresponding to the peptide sequence (DRPLTATEK) of an ORF52 (–10lgP 0f 44.48 and –1.6ppm), from F10:1136 detected by using the Bruker Impact II UHR-QqTOF 125 LC/MS system identified with PEAKS Studio software.

Using the MS methodology mentioned above, we detected all 25 previously reported virion proteins that were identified by Bechtel et al. [18] and Zhu et al. [19] (Table 1, Tables S1 and S4). We detected ORFs 18, 32, 38, 42, 43, 56, and K2, which were not identified as virion-associated in the first proteomic studies but have since been identified as virion proteins by other methods [20,21,22,23,24,25,26,27] (Table 2, Tables S2 and S5). Finally, we detected 10 KSHV proteins that have not been previously reported as virion associated. These novel virion proteins are ORFs 9, 23, 35, 48, 58, 72, K3, K9, K10, K10.5 (Table 3, Tables S3 and S6). Figure 3 shows an example of the protein coverage in our study (Figure 3) where a detected peptide sequence hit of ORF52 (DRPLTATEK) with –10lgp of 44.48 with an m/z of 515.7792, z = 2 using Collision-Induced Dissociation (CID) activation mode on qTOF detected Via PEAKS Studio software.

For some of the previously reported hits like ORF6 and ORF7, we were able to detect more unique peptide fragments than were reported initially; however, for some other proteins such as ORF25, the number of peptide hits we found was less than what was previously reported; Bechtel et al. [18] explain that one reason for having so many peptides for ORF25 in their study could be that virions were damaged during the purification and processing of their sample. Our reduced detection of ORF25 peptides could indicate that our altered sample preparation approach resulted in less damage to virions prior to protein extraction. 

Additionally, we detected ORF44 peptides only with chymotrypsin digestion and not with trypsin digestion, the peptide hits for this protein also followed our tight criteria of peptide mass tolerance of 20 ppm and fragment mass tolerance of 0.01; in addition to that, they all have −10lgP > 25. The identified peptides are included in Table S7.

It is important to note that we analyzed our dataset using a database containing only KSHV protein sequences, so our analysis does not include any host proteins associated with the virion. Next, we wanted to confirm that our novel hits (Table 3) were actually encapsulated within the virions and not due to debris that might have been coincidentally co-purified with the virion fraction. To accomplish this, we pre-treated intact, purified virions with 0.25% trypsin-EDTA to eliminate any proteins not being protected by the virion envelope before performing protein extraction and MS analysis (VTT protein fraction). We used a Western blot for gB to validate the trypsin pre-treatment’s efficacy in stripping the envelope layer (Figure 1c). Both original virion proteomic studies [18,19] identified beta-actin as a cellular protein incorporated into KSHV virions, so we utilized this marker as a loading control for this analysis. As expected, MS analysis of the digested pre-treated (VTT) protein fractions included all of the protein hits shown in Table 1, Table 2 and Table 3 except for 10 proteins, including the eight known glycoproteins ORFs 8, 22, 28, 39, 47, 53, 68, and K8.1, as well as ORF27 and one of our novel detected proteins, ORF58. Thus, we can conclude confidently that nine of our novel virion proteins, ORFs 9, 23, 35, 48, 72, K3, K9, K10, K10.5, are contained within the virion envelope and are not present in the sample due to contamination of the virion preparation with free proteins that are not indeed virion encapsulated. These results also support the prevailing hypothesis that ORFs 27 and 58 are glycoproteins [38,39,40].

**Figure 4:**
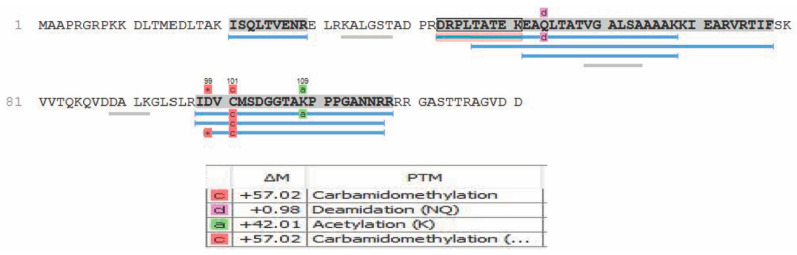
Example of post-translational modifications (PTMs) detected via PEAKS DB analysis for ORF52.

Moreover, these data reconfirm Zhu et al.’s [19] speculation that ORF28 is an envelope glycoprotein, based on only its similarity in homology to EBV BDLF3 protein. Of the proteins mentioned in Table 2, that have been introduced as tegument proteins of KSHV, ORF18 was reported by Gong et al. [22] to be essential for de novo lytic activity and viral reactivation, which is in line with our observation of this protein within the newly formed virions. Dai et al. [20] in 2014 reported ORF32 as a tegument assembly mediator. ORF42 has been reported to be an essential post-translational regulator, and the loss of it would result in the formation of fewer virion particles. ORF43 has been shown to function as a gate for the packaging of viral DNA into procapsids and a further injection of it to the host nucleus [25]. ORF56 plays a vital role in DNA replication and viral DNA synthesis and is reported to be a primary lytic gene. Interestingly, its protein expression levels have been reported to be dependent on ORF57 [26,41,42]. In our analysis, we also observed post-translational modifications including, but not limited to, oxidation, carbamidomethylation, deamidation, and acetylation. These modifications, where present, are indicated in the peptide sequences reported in Tables S1–S6. An example of some PTMs observed in the ORF52 protein are shown in Figure 4. It should be noted that, although our analysis was able to detect some PTMs and they are reported in our data, sample preparation for bottom-up proteomics often introduces modifications during sample preparation. Observation of the true spectrum of PTMs in KSHV virion proteins would require further studies using specialized methodologies that were not employed here.

## 4. Discussion

Since the emergence of system-wide protein analysis three decades ago, a common approach to proteomics has been the 2D separation of proteins followed by in-gel trypsinization of individual bands before sequencing and identification [43]. Despite its ease of use and flexibility, this technique has some significant drawbacks, including incomplete digestion, loss of sample, failure to detect low abundance proteins, and limited dynamic range for proteins. These limitations have resulted in a recent shift towards in-solution, gel-free, or “shotgun” proteomics [44]. Moreover, the development of ultra-high-pressure LC systems in conjunction with smaller inner diameter columns and particle size and development of algorithms and software for de novo sequencing has enabled the detection of thousands of proteins in single LC-MS runs [28,45]. Our study has taken advantage of these advances in proteomics technology to take a fresh look at the KSHV virion proteome.

The current study benefits majorly from a few advancements in KSHV research, some of which were not accessible at the time of the original studies done by Bechtel et al. [18] and Zhu et al. [19]. In particular, the iSLK cell producer cell line has greatly improved KSHV virion yields compared to previous systems isolating WT KSHV from PEL cell lines [35,46]. We believe this increased virion yield, combined with modern instrumentation and analysis software, contributed substantially to the increased sensitivity of our analysis.

EBV systems have shown that gamma-herpesvirus virions can have altered protein composition based upon the producer cell type [47]. However, differences in KSHV virion protein composition from different cell types have not been examined. Given that both original mass spectrometry studies analyzed KSHV virions from PEL cell lines, we cannot determine whether our novel proteins are a result of the increased sensitivity of our methods or the fact that these proteins are only incorporated into virions when they are produced in iSLK or possibly generally from cells of epithelial origin. Since the iSLK system has become increasingly prevalent in the field for the production of KSHV, these results provide critical insight into the protein composition of the iSLK-derived virions, which are commonly used for both in vitro and in vivo experimental studies of KSHV virology.

In addition to validating all of the previously reported proteins packaged within KSHV virions, we report 10 novel virion-associated proteins. ORF58 encodes a homolog of EBV BMRF2 protein with unknown functions [29], but together with ORF27, it has long been speculated to be a glycoprotein [38,39,40]. Our data that ORF58 is removed from the virion by pre-treatment of intact viral particles with trypsin support the conclusion that this ORF58 is a glycoprotein. Similarly, although ORF27 was not novel in our study, we showed that trypsin pre-treatment removed ORF27 from our virion proteome, thus confirming Zhu et al. [19] primary classification of ORF27 as a glycoprotein.

Three additional novel virion proteins detected here are highly conserved among gamma herpesviruses (ORF 23, 35, and 48). ORF23 binds to ORF34, and its specific function is still unknown [48]. However, a study in MHV68 has shown that its ortholog is not essential for in vitro and in vivo infection [49]. ORF35 encodes a poorly characterized protein that is essential for viral reactivation [50]. A study of MHV68 ORF35 has shown that it is essential for efficient lytic replication and latency [51]. ORF48 is also poorly studied in KSHV, but a study on the homologous protein in MHV68 has determined that this gamma-herpesvirus-conserved ORF is RTA-responsive and functions in both viral lytic replication and latency during in vivo infections [52]. Future studies into the functions of these proteins are needed to establish the implications of their presence in the virus particle.

ORF9 encodes the viral DNA polymerase [53] and interacts with K10/vIRF4 [25]. There is evidence of ORF9 gene expression as early as 0–10 hours post-infection [41], implying that genome replication is an important step early in the establishment of infection. ORF72 is the viral cyclin homolog, which shares 54% sequence homology with the cellular Cyclin D2 [54,55,56,57,58] and binds to a number of cellular cyclin-dependent kinases to promote proliferation of KSHV-infected cells [59,60,61] Packaging ORF9 and ORF72 into the virus particle implies that early genome replication and manipulation of the host cell cycle are critically important for the initial establishment of KSHV infection in a new host cell.

Our remaining novel virion proteins can broadly be classified as immunomodulatory and immune evasion genes. K2 expresses the viral IL-6 homolog [62], which has significant immunomodulatory effects during KSHV infection and KSHV-associated disease [63]. Although vIL-6 is known to signal intracellularly in the endoplasmic reticulum to manipulate host cell processes in an autocrine manner [64,65], we are unsure of the specific implications of the protein being contained in the virus particle where it would presumably be released into the cytoplasm upon infection. K3 is a membrane-associated ubiquitin ligase, which, along with K5, inhibits antigen presentation on Major Histocompatibility Complex Class I (MHC Class I) [66] and cell surface expression of receptors such as DC-SIGN [67]. Despite clear data from overexpression studies showing that K3 can reduce MHC-I expression at the cell surface [68], previous studies using K3 knockout viruses have led to the prevailing theory that K3 does not function in this way during lytic reactivation or early infection [46,69]. Importantly, however, the earliest timepoint examined by Brulois et al. in their 2012 study of de novo infection with a K3 knockout virus was 36 h post-infection, which is well past the window we would expect to see an effect of K3 as a virion-incorporated factor. Our data that K3 is virion incorporated establish a new context in which K3 may function very early in the viral life cycle (within hours of infection) and may provide an explanation for the previous negative data for K3 function in the context of viral infection.

Finally, we observed three viral interferon regulatory factor (vIRF) proteins as novel virion-associated proteins in our analysis. K9/vIRF-1 suppresses both type I and type II interferon responses [70,71]. Similarly, K10.5/vIRF-3 interferes with both type I and type II interferon responses and additionally can alter CIITA and MHC II expression [72,73]. In 2014, Lee et al. reported that K10/vIRF4 is a lytic protein capable of suppression of c-IRF4 and c-Myc, thus manipulating the host gene expression profiles to facilitate viral lytic replication [74]. In 2017, the same group characterized novel immune evasion strategies of vIRF4 to inhibit the IRF7-mediated IFN-α production [75,76]. It is not the first time that an immunomodulatory protein has been reported to be prepackaged within KSHV virion in order to have a quick response against immune recognition; ORF45, as reported by Zhu et al. [19,77], is not only packaged within the virion but is also known to interact with IRF7. Our data that three additional vIRFs are encapsulated within the virion necessitate further study into how these proteins may participate in immune evasion very early in KSHV infection.

## Figures and Tables

**Table 1:** Proteins identified in the VT fraction in our study that were previously reported by mass spectrometry.

Gene	Protein	aa Size	Previous Report by MS	Number of Unique Peptide Hits via Trypsin Digest	Number of Unique Peptide Hits via Chymotrypsin Digest
ORF6	Major DNA-binding protein (MBP)	1133	Zhu et al. [19]	7	6
ORF7	Tripartite terminase subunit 1 (TRM1)	695	Zhu et al. [19]	6	3
ORF8	Envelope glycoprotein B (gB)	845	Zhu et al. [19], Bechtel et al. [18]	10	20
ORF11	ORF11	407	Zhu et al. [19]	9	5
ORF17	Capsid Scaffolding protein	534	Zhu et al. [19]	3	3
ORF21	Thymidine kinase	580	Zhu et al. [19], Bechtel et al. [18]	8	3
ORF22	Envelope glycoprotein H (gH)	730	Zhu et al. [19], Bechtel et al. [18]	7	5
ORF24	ORF24	752	Bechtel et al. [18]	4	7
ORF25	Major capsid protein (MCP)	1376	Zhu et al. [19], Bechtel et al. [18]	5	6
ORF26	Triplex capsid protein 2 (TRX-2)	305	Zhu et al. [19], Bechtel et al. [18]	5	3
ORF27	ORF27	290	Zhu et al. [19]	6	4
ORF28	ORF28	102	Zhu et al. [19]	2	3
ORF33	Cytoplasmic envelopment protein 2 (CEP-2)	334	Zhu et al. [19], Bechtel et al. [18]	7	7
ORF39	Envelope glycoprotein M (gM)	400	Zhu et al. [19]	6	3
ORF45	ORF45	407	Zhu et al. [19]	5	4
ORF47	Envelope glycoprotein L (gL)	528	Zhu et al. [19]	5	3
ORF52	ORF52	131	Zhu et al. [19]	9	5
ORF53	Envelope glycoprotein N (gN)	110	Zhu et al. [19]	1	2
ORF62	Triplex capsid protein 1 (TRX-1)	331	Zhu et al. [19], Bechtel et al. [18]	4	3
ORF63	Inner tegument protein	927	Zhu et al. [19], Bechtel et al. [18]	7	5
ORF64	Large tegument protein deneddylase	2635	Zhu et al. [19]	6	12
ORF65	Small capsomere-interacting protein (SCP)	170	Zhu et al. [19]	4	3
ORF68	Packaging protein UL32 homolog	545	Zhu et al. [19]	3	4
ORF75	ORF75	1296	Zhu et al. [19], Bechtel et al. [18]	8	6
ORF K8.1	gp35/37	228	Zhu et al. [19]	4	4

Proteins identified in VT fraction of our study that were previously reported by mass spectrometry. All peptides have a score (−10lgP) greater than 25 and correspond to a statistical significance of *p* < 0.00316; moreover, from our positive matches, only proteins identified with more than at least 3 peptide sequences with a mass tolerance of <0.05 Da were reported.

**Table 2:** Proteins identified by mass spectrometry in our study that were previously reported by other methods.

Gene	Protein	aa Size	Previous Report by MS	Unique Peptide Hits via Trypsin Digest	Unique Peptide Hits via Chymotrypsin Digest
ORF18	Protein UL79 homolog	257	Gong et al. [22]	4	4
ORF32	Capsid vertex component 1 (CVC-1)	454	Dai et al. [20]	7	4
ORF38	Cytoplasmic envelopment protein 3 (CEP-3)	61	Wu et al. [21]	4	3
ORF42	Cytoplasmic envelopment protein 1 (CEP-1)	278	Butnaru et al. [23]	3	3
ORF43	Portal Protein	605	Dünn-Kittenplon et al. [24]	6	5
ORF48	ORF48	402	Sander et al. [25]	6	3
ORF56	DNA primase	843	Majerciak. [26]	6	4
ORF K2	Viral Interleukin 6 Homolog	204	Katano, H. et al. [37]	12	7

Proteins identified in VTT fraction of our study that were previously reported by non-mass spectrometry methods. All peptides have a score (−10lgP) greater than 25 and correspond to a statistical significance of *p* < 0.00316; moreover, from our positive matches, only proteins identified with more than at least 2 peptide sequences with a mass tolerance of <0.05 Da were reported.

**Table 3:** Virion-associated proteins identified that have not previously been reported by any method.

Gene	Protein	aa Size	Unique Peptide Hits via Trypsin Digest	−10lgP (Trypsin)	Unique Peptide Hits via Chymotrypsin Digest	−10lgP (Chymotrypsin)
ORF9	DPOL	1012	6	45.15	4	34.08
ORF23	ORF23	404	6	26.45	4	32.21
ORF35	ORF35	150	4	26.23	3	37.48
ORF58	ORF58	357	5	42.45	4	41.66
ORF72	viral cyclin homolog	257	4	42.46	4	45.19
ORF K3	E3 ubiquitin-protein ligase MIR1	333	5	35.01	3	32.7
vIRF-1	Viral IRF-like protein 1	449	5	39.12	4	26.26
vIRF-3	Viral IRF-like protein 3	566	7	27.51	3	26.50
vIRF-4	Viral IRF-like protein 4	911	11	37.95	5	30.02
ORF44	DNA Replication helicase	788	0	N/A	5	35.06

Novel proteins identified in VTT fraction. All peptides have a score (−10lgP) greater than 25 and correspond to a statistical significance of *p* < 0.00316; moreover, from our positive matches, only proteins identified with more than at least 2 peptide sequences with a mass tolerance of <0.05 Da were reported.

## Supplementary Materials

The following are available online at https://www.mdpi.com/1999-4915/12/12/1382/s1, Table S1: Protein hits reported previously by other labs through Mass-Spectrometry and detected in our analysis as well using Trypsin as digesting enzyme including the peptide sequences detected in our study. Table S2: Protein hits unique to our mass spectrometry but reported previously through other methods using Trypsin as digesting enzyme including the peptide sequences detected in our study, as well as −10logP value of each sequence. Table S3: Protein hits uniquely detected in our Study using Trypsin as digesting enzyme including the peptide sequences detected in our study, as well as −10logP value of each sequence. Table S4: Protein hits reported previously by other labs through Mass-Spectrometry and detected in our analysis as well using Chymotrypsin as digesting enzyme including the peptide sequences detected in our study. Table S5: Protein hits unique to our mass spectrometry but reported previously through other methods using Chymotrypsin as digesting enzyme including the peptide sequences detected in our study, as well as −10logP value of each sequence. Table S6: Protein hits uniquely detected in our Study using Chymotrypsin as digesting enzyme including the peptide sequences detected in our study, as well as −10logP value of each sequence. Table S7: Protein hits uniquely detected in our Study using Chymotrypsin as digesting enzyme including the peptide sequences detected in our study, as well as −10logP value of each sequence, but not found in Trypsin digested fraction. Table S8: Example of de novo peptide sequence hits detected in our study via implementation of de novo sequencing feature of PEAKS DB with cut-off ALC% at 75.
