# Supplementary material for: An Update of the Virion Proteome of Kaposi Sarcoma-Associated Herpesvirus
Source: Viruses. 2020 Dec 2;12(12):1382. doi: 10.3390/v12121382 (PMC7761624; doi:10.3390/v12121382)
Supplement: Supplementary file 1 [file viruses-12-01382-s001.pdf]

Supplementary Tables

Table S1: Hits reported previously by other labs through Mass-Spectrometry and detected in our analysis as well using Trypsin as digesting enzyme

Gene	Protein	Amino-Acid	Number of Unique Peptide Hits	Sequences of Correspondence detected
ORF6	Major DNA-binding protein (MBP)	1133	7	244-251: R.QFVHDQYK.I<br>569-587: K.GIDSTVEAELLKFINC(+57.02)M(+15.99)IK.N<br>691-712: F.C(+57.02)DLFDTDAAIGGMFAPARMQVR.I<br>43-49: G.NGYPEAK.V<br>574-580: T.VEAELLK.F<br>740-752: E.SIQAGFMKPASQR.D<br>1012-1031: R.DNPNLPKTVVLELVKHLGSS.C
ORF7	Tripartite terminase subunit 1 (TRM1)	695	6	327-355: R.GTPKHFFDC(+57.02)FRPDSLETLFC(+57.02)GGLFSSVED.T<br>546-568: L.FPSPPNVTLAQC(+57.02)FEAAGMLPHQK.M<br>23-33: L.SYADPATLDTK.S<br>414-426: R.QLVGGDKPEEVLR.D<br>355-362: L.TPESC(+57.02)VKA.C<br>454-470: A.SAGLFTFQPLLSNSTHR.K
ORF8	Envelope glycoprotein B (gB)	845	10	650-662: R.LASSVFDLETMFR.E<br>834-845: L.TQSLDISPETGE<br>197-215: R.YFSQPVIYAEPGWFPGIYR.V<br>692-705: R.DLSEIVADLGGIGK.T<br>163-173: K.VNVNGVENTFT.D<br>778-788: P.SGGAPTREEIK.N<br>581-588: T.NNQVETC(+57.02)K.D<br>70-78: S.ASITGELFR.F<br>674-686: G.LREDLDNTIDMNK.E<br>165-175: N.VNGVENTFTDR.D
ORF11	ORF11	407	9	24-42: R.GEPVWDSVIHPSHIVISNR.V<br>67-78: R.AYPNFTFDNTHR.K<br>118-147: R.ASVSANIAVGGLKIIILALTLVHAQGVYLR.C<br>151-164: K.DLSTPHC(+57.02)APAIVQR.E<br>165-195: R.EVLSSGFEPQFTVTGIPVTSSNLNQC(+57.02)YFLVR.K<br>230-261: R.ISVTAPAQETPVWGLVTTSFSLTPTAPLAFDR.N<br>275-285: R.HYIPVIYSGPK.I<br>294-309: R.QVVWHNNSYTSSLPC(+57.02)K.V<br>310-339: K.VTAIVSNHC(+57.02)C(+57.02)NC(+57.02)DIFLEDSEWRPNKPAPLK.L
ORF17	Capsid Scaffolding Protein (CSP)	534	3	386-397: W.HAGPPPSSSAAA.A<br>421-433: K.EHGGTYVHPPIYV.Q<br>264-285: F.LSMLQSSIDGMKTTAAKMSHTL.S
ORF21	Thymidine kinase	580	8	382-395: R.LSFDHFFQLLSIFR.A<br>486-504: K.NLHEQSMLPMITGVLDPVR.H<br>364-373: R.HLLSPAVVFP.L<br>252-264: R.NVYLLYLEGVM(+15.99)GV.G<br>274-294: V.C(+57.02)GILPQERVTSFPEPMVYWTR.A<br>328-354: K.FSLPFRTNATAILRMMQPWNVGGGSGR.G<br>382-392: R.LSFDHFFQLLS.I<br>244-251: R.TPVTVDYR.N
ORF22	Envelope glycoprotein H (gH)	730	7	565-597: K.IIATVPLPHVTYIISSEALSNAVVYEVSEIFLK.S<br>154-163: I.FASKWSLFAR.D<br>164-184: R.DTPEYRVFYPMNVM(+15.99)AVKFSIS.I<br>661-668: R.VQTNLFLD.K<br>629-636: S.TPRRGC(+57.02)PL.C<br>158-169: K.WSLFARDTPEYR.V<br>165-210: D.TPEYRVFYPMNVM(+15.99)AVKFSISIGNNESGVALYGVVSEDFVVVTLHNR.S

ORF24	ORF24	752	4	395-412: T.DARTLGSSTVSDMLEPTK.H<br>86-96: Q.EGTLLGKVGRR.Y<br>422-444: I.TIFNTNM(+15.99)VINTKISC(+57.02)HVPNTLQK.T<br>372-391: Y.APKDRRAAMKGNLQAC(+57.02)FQRY.A
ORF25	Major capsid protein (MCP)	1376	5	123-143: HHIGAEIELAAADIELLFAEK<br>158-172: TITSALQFGMDALER<br>1057-1070: ASTSMFVGLPSVVR<br>1243-1255: R.GSLGDVLYNITFR.Q<br>1293-1335: R.LAGAPATSTTDLQYVVVNGTDVFLDQPC(+57.02)HMLQEAYPTLAASHR.V
ORF26	Triplex capsid protein 2 (TRX-2)	305	5	203-220: R.VLDDLSMYLC(+57.02)ILSALVPR.G<br>268-285: R.VMFSYLQSLSSIFNLGPR.L<br>90-96: P.SDN(+.98)LQIK.N<br>232-251: R.HDRHPLTEVFEGVVPDEVTR.I<br>175-189: L.DLYTTNVSFM(+15.99)GRTYR.L
ORF27	ORF27	290	6	1-26: M(+15.99)ASSDILSVARTDDGSVC(+57.02)EVSLRGGR.K<br>169-189: Y.SM(+15.99)ALRRAFAVMVNTSC(+57.02)AGVTL.C<br>104-110: V.TPSSIEF.A<br>44-60: T.DAIKDAFLSDGIVDMAR.K<br>35-60: P.DTEPWVVETDAIKDAFLSDGIVDM(+15.99)AR.K<br>179-191: V.MVNTSC(+57.02)AGVTLC(+57.02)R.G
ORF28	ORF28	102	2	63-73: ATVAYQVLR<br>74-96: TLGPQAGSHAPPTVGIATQEPYR
ORF33	Cytoplasmic envelopment protein 2 (CEP-2)	334	7	117-126: R.FPYIAPPPSR.E<br>117-145: R.FPYIAPPPSREHVPDLTRQELVHTSQVVR.R<br>135-145: R.QELVHTSQVVR.R<br>160-196: R.NVNPFVWLGGGSVWLLFLGVDYMAFC(+57.02)PGVDGMPSLAR.V<br>257-270: R.NFLGLLFDPIVQSR.V<br>276-303: K.ITSHPTPTHVENVLTGVLDDGTLVPSSK.A<br>318-324: R.LLIYEC(+57.02)K.K
ORF39	Envelope glycoprotein M (gM)	400	6	6- 16: SDRFLMSSWVK<br>105-149: AKHVHATTMMSM(+15.99)QSWIALLGSHSVLYVAILRM(+15.99)WSM(+15.99)QLFIHVLSYK<br>136-149: M(+15.99)WSMQLFIHVLSYK<br>270-290: QVGFYVGVFVGYLILLLPVIR<br>370-379: VKDISTPAPR<br>380-400: TQYQSDHESDSEIDETQMIFI
ORF45	ORF45	407	5	266-288: HFSHQPPSSEEDGEDQGEVLSQR<br>356-381: GHLPTQSPSTSAHSISSGSTTTAGSR<br>17-25: R.MLPIEGAPR.R<br>201-218: Y.GLSPASRNSVPGTQSSPY.S<br>50-68: R.GYAGPTVIDMSAPDDVFAE.D
ORF47	Envelope glycoprotein L (gL)	528	5	17-25: MLPIEGAPR<br>121-137: AMAMFVRTSSSTHDEER<br>121-146: AM(+15.99)AM(+15.99)FVRTSSSTHDEERM(+15.99)LPIEGAPR<br>387-409: HFSHQPPSSEEDGEDQGEVLSQR<br>477-503: GHLPTQSPSTSAHSISSGSTTTAGSR
ORF52	ORF52	131	9	98-117: R.IDVC(+57.02)MSDGGTAKPPPGANNR.R<br>47-78: L.TATEKEAQLTATVGALSAAAAKKIEARVRTIF.S<br>52-68: K.EAQLTATVGALSAAAAK.K<br>43-51: R.DRPLTATEK.E<br>21-29: K.ISQLTVENR.E<br>99-117: I.D(+57.02)(+57.02)VC(+57.02)MSDGGTAKPPPGANNR.R<br>98-117: R.IDVC(+57.02)MSDGGTAKPPPGAN(+.98)NR.R<br>98-118: R.IDVC(+57.02)MSDGGTAK(+42.01)PPPGANNRR.R<br>52-68: K.EAQ(+.98)LTATVGALSAAAAK.K
ORF53	Envelope glycoprotein N (gN)	110	1	103-110: FVDEVVHA
ORF62	Triplex capsid protein 1 (TRX-1)	331	4	45-53: K.YAASTRPTV.G<br>211-217: S.C(+57.02)PMVQRR.E<br>4-10: V.QAENAAR.L<br>68-73: R.QPTYGDFLVYSQTFSPQ.E

ORF63	Inner tegument protein	927	7	445-452: R.ALAFFVPP.A<br>480-491: K.TTWGGAVPANLA.R<br>501-512: T.QHISSTPPPTLK.D<br>604-624: R.HSQDALYNLLDC(+57.02)IQELFTHIR.Q<br>725-741: R.EATVAMTTIAKPIYPAY.I<br>461-495: R.VYAALPSQLM(+15.99)RAIFEISVKTTWGGAVPANLARDID.T<br>748-781: R.LEYLNRLNHHILRIPFPQDALSELQETYLAAFAR.L
ORF64	Large tegument protein deneddylase	2635	6	129-135: R.NYAGTVQ.Y<br>1979-1985: M.EFGPPPK(+42.01).T<br>384-406: R.EPPPTPPATPGATALLSDLTATR.G<br>2358-2365: R.T(+75.98)ALQPPRT.E<br>2428-2445: R.DTS(+238.23)PPAEKRAAPVIRVMA.P<br>1265-1273: Y.Q(+57.02)D(+57.02)QVSFFLR.T
ORF65	Small capsomere-interacting protein (SCP)	170	4	8-14: DPVIQER<br>28-45: M(+15.99)NTLDQGNM(+15.99)SQAEYLVQK<br>28-46: M(+15.99)NTLDQGNMSQAEYLVQKR<br>47-64:HYLVFLIAHHYYETYLRR
ORF68	Packaging protein UL32 homolog	545	3	290-324: K.NGTASVC(+57.02)LLC(+57.02)EC(+57.02)LAAHPEAPKALQTLQC(+57.02)EVMGHIE.N<br>454- 467: LDLAHPSQTSHLYA<br>5-15: K.EPSAVHPDAPR.I
ORF75	ORF75	1296	8	63-84: DVEIQTVLAVLSPLLGYPHVIR<br>139-153: K.ITQTLLEPHPPQFIR.A<br>442-495: R.AHLPADPAAGPDAVEAAVAEHFLNVYC(+57.02)SLVFAVVAESGAVPGDLGETPLEVLQR.A<br>552-575: R.VSGHPEDVDWGLFATGSTIHQLLR.H<br>630-647: R.VSGHPEDVDWGLFATGSTIHQLLR.H<br>792-799: R.YRVTPDVK.V<br>1206-1224: R.GEITLTYHGNAADETLPAR.H<br>1229-1245: R.NPTGNSTVAGLTSSDGR.H
K8.1	gp35/37	228	4	33-53: SHLGFWQEGWSGQVYQDWLGR<br>65-87: L.EAVSLNGTRLAAGSPSSEYPNVS.V<br>137-155: L.ISAFSGSYSSGEPSRTTRI.R<br>173-183: VPFSATTTTTR

Table S2: Hits unique to our mass spectrometry but reported previously through other methods using Trypsin as digesting enzyme

Name	Amino Acid Size	Protein detected	Reported previously by other methods	Number of unique peptide hits	Sequences of Correspondence detected	-10lgP
ORF18	257	Protein UL79 homolog	Gong et al 2014	4	22-32: S.LEMRLVTMC(+57.02)VK.E<br>47-57: M(+15.99)YNFGLNVYLLR<br>119-140: K.LSVGREAVYLHVGLSERGRFLT.L<br>148-159: LFNLGSVLPC(+57.02)R	28.41<br>41.6<br>27.45<br>33.37
ORF32	454	Capsid vertex component 1 (CVC-1)	Dai et al 2014	7	224-232: R.LSFNPVNAD.V<br>149-167: S.VLDARDTPGFRARPLPTSR.D<br>274-293: E.EPVPPPPGLVFM(+15.99)DDLFINTK.Q<br>301-313: T.LEAAC(+57.02)RTQGYTLR.Q<br>133-146: R.SPTVDGVSPPEGAV.A<br>102-155:<br>R.ISC(+57.02)PGSNLSLTVRFLYLSLVVAMGAGRNNARSPTVDGVSPPEGAVAHPLEELQR.L<br>10-17: R.YVGPRC(+57.02)HR.L	37.48<br>37.41<br>35.18<br>28.43<br>28.27<br><br>27.63<br>25.1
ORF38	61	Cytoplasmic envelopment protein 3 (CEP-3)	Wu et al 2016	4	10-32: RPSQPVDVDGEPLDVVVDYDPIR<br>33-51: VSEKGM(+15.99)LLEQSQSPYPALK<br>37-51: GMLLEQSQSPYPALK<br>20-36: G.EPLDVVVDYDPIRVSEK.G	44.09<br>25<br>25<br>27.74
ORF42	278	Cytoplasmic envelopment protein 1 (CEP-1)	Butnaru et al 2019	3	35-42:K.GNGELMMR.A<br>170-189: I.ETLTATAAFVYELSVDDHFR.A<br>9-21: A.RLTGVPM(+15.99)STHAPK.T	32.87<br>27.17<br>25.6
ORF43	605	Portal Protein	DD Dünn-Kittenplon et al, 2019	6	480-506: E.SHLGSSSYC(+57.02)YMC(+57.02)SDSAINTANIYC(+57.02)LIR.Y<br>464-479:K.DLTNLWESEMFQTYKL.A<br>555-565:L.VTPPFENVPGK.G<br>44-54:L.RNPGVFFRQLF.I<br>156-163:L.VNSIQEQL.M<br>377-385:L.RKDTC(+57.02)SMSL.A	27.15<br>33.22<br>30.47<br>28.11<br>27.03<br>40.1
ORF48	402	ORF48	Sander,G. et al .2007	6	336-343:D.VTLVHTAR.M<br>192-222:V.EFRRELSLISSC(+57.02)LNVC(+57.02)WLYHIFIEHITSDVR.R<br>214-222:F.IEHITSDVR.R<br>313-321:R.LDLSHFDRR.R<br>362-384:K.VSLLHIC(+57.02)SYSMEADVPVPGQQLN.T<br>190-201:R.M(+15.99)ESTRIIGAC(+57.02)PF.A	25.76<br>37.34<br>35.49<br>36.05<br>32.42<br>31.38
ORF56	843	DNA primase	Davis et al 2015	6	525-538: R.EAILDIIQLLGPVD.P<br>497-507: R.WVLDFDLPVC(+57.02)R.D<br>535-548:L.GPVDPRTHPVYFFK.S<br>426-439:K.QFFTMLQEDGLERY.W<br>595-605:V.ALTGILNRTIK.L<br>701-708:R.NENFLENK.T	27.05<br>25<br>25.67<br>25.14<br>27.35<br>25.31
ORF K2	204	Viral Interleukin 6 Homolog	Katano,H. et al.2000	12	33-56: K.DLLIQRLNWMLWVIDEC(+57.02)FRDLC(+57.02)YR.T<br>39-51:R.LNWMLWVIDEC(+57.02)FR.D<br>62-73:K.GILEPAAIFHLK.L<br>62-96:K.GILEPAAIFHLKLPAINDTDHC(+57.02)GLIGFNETSC(+57.02)LKK.L<br>96-117:K.KLADGFFEFEVLFKFLTTEFGK.S<br>96-109: K.KLADGFFEFEVLFK.F<br>97-117:K.LADGFFEFEVLFKFLTTEFGK.S<br>97-109::K.LADGFFEFEVLFK.F<br>118-130::K.SVINVDVMELLTK.T<br>131-142:K.TLGWDIQEELNK.L<br>166-182:K.YWVRHFASFYVLSAMEK.F<br>170-182:R.HFASFYVLSAMEK.F	48.85<br>66.32<br>40.95<br>25.72<br>64.76<br>47.06<br>25.94<br>25.66<br>50.54<br>47.99<br>27.17<br>51.43

Table S3: Hits unique to our Study using Trypsin as digesting enzyme

Name	Amino Acid Size	Protein detected	Number of unique peptide hits	Sequences of Correspondence detected	-10lgP
ORF9	1012	DPOL	6	69-82: K.RGEVFAGETGSIWK.T<br>70-82: R.GEVFAGETGSIWK.T<br>396-404: Y.DFKLQDFTK.I<br>470-482: K.DDISYKDIPPLFK.S<br>671-677: K.TILDKQQ.L<br>938-950: R.IPYVFVDAPGSLR.S	26.31<br>26.87<br>44.45<br>26.41<br>45.15<br>30.99
ORF23	404	ORF23	6	30-67: T.SPAVAAMVGVSNPEVPMPLLFEKFGTPDSSTLPLYAAR.H<br>356-389: Q.SAAWLGAGVVELIC(+57.02)DGNPLSEVLGFLAKYMPIQK.E<br>225-251: R.LGESPVC(+57.02)DFNTVTIM(+15.99)ERANNSITFLPK.L<br>354-360: D.RQSAAWL.G<br>193-215: H.NIAQVC(+57.02)ERDIVSLNTDNEAASMF.Y<br>269-291: R.SMGLENIVSC(+57.02)FSSLYGAELAPAK.T	31.71<br>59.54<br>40.2<br>36.59<br>72.52<br>33.22
ORF35	150	ORF35	4	94-105: D.IDALVDAVADLK.E<br>87-102: L.KSASNAEEPLIASEPL.A<br>45-52: R.NLASPDHV.R<br>120-130: L.TFSAVGVLHTD.G	29.35<br>43.54<br>28.98<br>27.76
ORF58	357	ORF58	5	54-66: W.DVEFFRLVAAPVF.K<br>32-38: P.AYFGSVL.V<br>30-42: I.FPAYFGSVLVALR.T<br>101-120: R.VPFIPGLC(+57.02)VLNC(+57.02)LLLLPYPL.A<br>172-178: V.GGLLAFR.H	28.43<br>28.21<br>32.22<br>28.78<br>25.43
ORF72	257	viral cyclin homolog	4	153-160: A.IESGLYDR.L<br>146-154: R.TEAVLATDV.T<br>21-44: R.IFYNILEIEPRFLTSDSVFGTFQQ.S<br>54-82: LLGTWMFSVC(+57.02)HEYNLEPNVVALALNLLDR	35.76<br>32.47<br>26.09<br>25.94
ORF K3	333	E3 ubiquitin-protein ligase MIR1	5	2-35:<br>EDEDVPVC(+57.02)WIC(+57.02)NEELGNERFRAC(+57.02)GC(+57.02)TGELENVHR<br>26-51: AGPSSLVDM(+15.99)LPQGLPGGGYGSMGVIR<br>250-271: DDNVEPTAVGC(+57.02)DC(+57.02)NNLGAERYR<br>308-321: Q.GLPGGGYGSMGVIR.K<br>279-292: Y.VGAQSGDGAYSVSC(+57.02).H	25.06<br><br>26<br>25<br>42.27<br>29.5
vIRF-1	449	Viral IRF like protein 1	5	246-281: T.AEGQEAVIDWGRLFIRM(+15.99)YYNGEQVHELLTTSQSGC(+57.02)R.I<br>94-110: K.DWIVC(+57.02)QVNSGKFPGVEW.E<br>92-117: S.IKDWIVC(+57.02)QVNSGKFPGVEWEDEERTR.F<br>26-67:<br>F.KAWSVGATRNVPMGAGRGGGPC(+57.02)LC(+57.02)ARNINTKTPFPTPTAVEW.C<br>52-58: R.NINTKTP.F	30<br>25.33<br>25.21<br>33.62<br>30.86
vIRF-3	566	Viral IRF like protein 3	7	72-80: L.GSPITAFGK.I<br>128-136: L.HSGSSLWEI.L<br>30-52: K.ASEVC(+57.02)AADVSGVPRPADMTEPTK.L<br>559-566: R.ENVLSSSP<br>478-490: R.MEVPLSFRPEEWR.V<br>15-24: L.RSASGIASGL.D<br>35-52: C.AADVSGVPRPADMTEPTK.L	31.89<br>25.72<br>32.53<br>31.04<br>27.9<br>26.18<br>25.26

vIRF-4	911	Viral IRF like protein 4	11	505-547: T.QGGASATPSAGAPPTPEVAERQEPSSSGIPYVC(+57.02)QGDNM(+15.99)ATGYR.R<br>752-785: EGDAGEAMLC(+57.02)SWPVGDTLGHLC(+57.02)QSFVPELLRIPR<br>152-158: R.RSDTREQ.S<br>435-467: F.EPQPPSAPAPGYAKPSC(+57.02)YNWSPLAEPPATRPIR.A<br>161-175: S.APAHRPPSPLTWLWR.T<br>549-568: R.VTTSSGALEVEIIDLTGDSD.T<br>858-868: R.YLATTAAIPQT.P<br>677-696: G.NILSELQEEPSSSTRQATDR.R<br>825-836: Q.VEGWWFGNPNSR.Y<br>492-501: W.SSGGAPNQGL.S<br>407-421: R.ETGAEGAC(+57.02)GASTEGR.A	30.84<br>26.13<br>37.95<br>25<br>53.33<br>44.03<br>37.87<br>36.42<br>34.83<br>29.55<br>28.06

Table S4: Hits reported previously by other labs through Mass-Spectrometry and detected in our analysis as well using Chymotrypsin as digesting enzyme

Gene	Protein	Amino-Acid	Previous Report by Mass-Spectroscopy	Number of Unique Peptide Hits	Sequences of Correspondence detected
ORF6	Major DNA-binding protein (MBP)	1133	Zhu et al 2005	6	77-88: I.DATTASVKLTSY.H<br>111-120: L.EKLC(+57.02)RESREL.F<br>627-650: L.LTVIQDIC(+57.02)LTSC(+57.02)M(+15.99)MYEQDNPAVGI.V<br>755-761: S.YIVGGPY.M<br>984-990: L.VKRIVGI.N<br>961-968: F.KKNNVSSM.L
ORF7	Tripartite terminase subunit 1 (TRM1)	695	Zhu et al 2005	3	31-37: L.DTKSLAL.T<br>99-113: L.LHLDITC(+57.02)NKHRSVRF.N<br>666-672: L.VDKKYGW.I
ORF8	Envelope glycoprotein B (gB)	845	Zhu et al 2005, Bechtel et al 2005	20	57-63: G.PKSVDFY.Q<br>63-77: F.Y(+79.97)QFRVC(+57.02)SASIT(+79.97)GELF.R<br>67-76: R.VC(+57.02)SASITGEL.F<br>82-92: L.EQTC(+57.02)PDTK(+42.02)DKY.H<br>82-92: L.EQTC(+57.02)PDTKDKY.H<br>93-105: Y.HQEGILLVYKKNI.V<br>117-125: R.KIATSVTVY(+6.01).R<br>138-155: Y.ELPRPVPLYEISHMDSTY.Q<br>147-158: Y.E(+57.02)(+57.02)ISH(+57.02)MDSTYQC(+57.02)F.S<br>202-209: P.V(+42.01)IYAEPGW.F<br>275-281: L.NHTVVTY.S<br>287-295: T.SPTPQN(+203.08)RIF.V<br>364-371: L.TSDINTTL.N<br>377-390: A.KLASTHVPNGTVQY.F<br>392-398: F.HT(+27.05)TGGLY.L<br>402-408: W.QPMSAIN(+14.02).L<br>466-473: Y.DKLRDGIN.Q<br>469-476: L.R(+27.99)DGINQVL.E<br>511-518: Y.GRPVS(-18.01)AKF.V<br>519-548: F.VGDAISVTEC(+57.02)INVDQSSVNIHKSLRTNSKD.V<br>532-555: V.DQSSVNIHKSLRTNSKDVC(+57.02)YARPL.V<br>534-542: Q.S(+28.03)SVNIHKSL.R

					572-579: L.GARNEIIL.T<br>588-594: C.KDTC(+57.02)E(+57.02)HY.F<br>651-657: L.ASSVFDL.E<br>675-689: L.REDLDNTID(+1572.99)MNKERF.V<br>726-739: F.INFIKHPLGGMLMI.I<br>734-740: L.GGMLMII.I<br>738-747: L.M(+15.99)IIIVIAIIL.I<br>751-759: F.M(+31.99)LSRRTNTI.A<br>767-791: M.IYPDVDRRAPPSGGAPTREEIKNIL.L<br>809-820: L.KKS(+162.05)TPSVFQRTA.N<br>817-823: F.Q(+42.01)(+.98)RTANGL.R
ORF11	ORF11	407	Zhu et al 2005	5	13-32: F.AVC(+57.02)SSKRQLGRGEPVWDSVI.H<br>65-73: L.HRAYPNFTF.D<br>166-175: E.VLSSGFEPQF.T<br>194-201: L.VRKPKSRL.A<br>229-242: L.RISVTAPAQETPVW.G
ORF17	Capsid Scaffolding Protein (CSP)	534	Zhu et al 2005	3	64-91: H.GIFC(+57.02)TGAITSPAFLELASRLADTSHVAR.A<br>181-197: R.LEDLSTPNFVSPLETLM(+15.99).A<br>183-199: E.DLSTPNFVSPLETLM(+15.99)AK.A
ORF21	Thymidine kinase	580	Zhu et al 2005, Bechtel et al 2005	3	96-106: L.IRHPSEKGSIF.A<br>129-167:<br>F.QSPRVC(+57.02)GRPPLPPPNHPPPATRPADASM(+15.99)GDVGWADLQGL.K<br>392-401: L.SIFRATEGDV.V
ORF22	Envelope glycoprotein H (gH)	730	Zhu et al 2005, Bechtel et al 2005	5	233-241: L.KGHATYDEL.T<br>315-321: F.QMLVAHF.L<br>584-601: L.SNAVVYEVSEIFLKSAMF.I<br>597-614: L.KSAMFISAIKPDC(+57.02)SGFNF.S<br>629-636: S.TPRRGC(+57.02)PL.C
ORF24	ORF24	752	Bechtel et al 2005	7	127-139: S.WELTDDC(+57.02)DKPC(+57.02)EF.R<br>172-179: W.EFEQC(+57.02)FHA.F<br>319-326: L.VYPGTPAI.Y<br>372-391:Y.APKDRRAAMKGNLQAC(+57.02)FQRY.A<br>485-493: I.NINISGDML.H<br>543-557: F.WTTNFPSVVSSKDGL.N<br>709-715: F.AALKPQI.V
ORF25	Major capsid protein (MCP)	1376	Zhu et al 2005, Bechtel et al 2005	6	547-554: P.C(+57.02)PGARGSY.R<br>836-845: Y.NGPVFADVVN.A<br>860-868: L.KDILQAGDI.R<br>1063-1086: F.VGLPSVVRREVRSDAVTFEITHEI.A<br>1316-1337: F.LDQPC(+57.02)HMLQEAYPTLAASHRVM(+15.99).L<br>1342-1349: Y.MSNKQTHA.P
ORF26	Triplex capsid protein 2 (TRX-2)	305	Zhu et al 2005, Bechtel et al 2005	3	72-87: AVLEEVRPDSLRLTRM<br>156-171: HIYSKISAGAPDDVNM<br>238-266: TEVFEGVVPDEVTRIDLDQL SVPDDITRM
ORF27	ORF27	290	Zhu et al 2005,	4	24-32: R.GGRKKTTVY.L<br>45-51: D.AIKDAFL.S<br>52-72: L.SDGIVDM(+15.99)ARKLHRGALPSNSH.N<br>104-110: V.TPSSIEF.A
ORF28	ORF28	102	Zhu et al 2005	3	1-13: MSMTSPSPVTGGM<br>39-52: VIGACVYCCIRVFL<br>76-102: GPQAGSHAPPTVGIATQEPY RTIYMPD
ORF33	Cytoplasmic envelopment protein 2 (CEP-2)	334	Zhu et al 2005 , Bechtel et al 2005	7	35-51: C.TSISPVY(+79.97)DPELVTSYAL.S<br>47-56: V.TSYALSVPAY.N<br>50-64: Y.A(+57.02)LSVPAYN(+.98)VSVAILL.H<br>138-144: L.VHTSQVV.R<br>185-194: F.C(+57.02)P(+31.99)GVDGMPSL.A<br>234-240: L.SNIC(+57.02)PC(+57.02)I.K<br>291-298: T.GVLDDGTL.V
ORF39	Envelope glycoprotein M (gM)	400	Zhu et al 2005	3	1-8: M(+15.99)RASKSDR.F<br>58-66: L.TVRNSAKHL.T<br>367-400: A.RAKVKDISTPAPRTQYQSDHESDSEIDETQMIFI

ORF45	ORF45	407	Zhu et al 2005	4	18-45: LPIEGAPRRRPPVKFIFPPP PLSSLPGF<br>94-121: DEDEEDEDEEENDDVQEEDE PEGYPADF<br>293-395: DVGQKRKRQSTASSGSEDVV RCQRQPNLSRKAVASVIIIS SGSDTDEEPSSAVSVIVSPS STKGHLPTQSPSTSAHSISS GSTTTAGSRCSDPTRILAST PPL<br>321-395: SRKAVASVIIISSGSDTDEE PSSAVSVIVSPSSTKGHLPT QSPSTSAHSISSGSTTTAGS RCSDPTRILASTPPL
ORF47	Envelope glycoprotein L (gL)	528	Zhu et al 2005	3	22-39: VALPCCAIQASAASTLPL<br>114-140: TVGFNATTADSSIHNVNIII ISVGKAM<br>141-167: NRTGSVSGSQTRAKSSSRRA HAGQKGK
ORF52	ORF52	131	Zhu et al 2005	5	18-24: L.T(+79.97)AKISQ(+.98)L.T<br>25-31: L.TVENREL.R<br>32-41: L.RKALGSTADP.R<br>36-42: L.GSTAD(+57.02)PR.D<br>97-131: L.RIDVC(+57.02)MSDGGTAK(+14.02)PPPGANNRRRRGASTTR(+14.02)AGVDD
ORF53	Envelope glycoprotein N (gN)	110	Zhu et al 2005	2	18-42: HCWVTANSTGVASSTERSSP STAG.L<br>52-58: T.SVTTPGF.Y
ORF62	Triplex capsid protein 1 (TRX-1)	331	Zhu et al 2005, Bechtel et al 2005	3	46-65: AASTRPTVGSLHEALRQAPF<br>244-263: L.TFFQSGKGFAEVVAM(+15.99)IKDHF.T<br>297-315: DSHPVHQSLNVKGTSLPVL
ORF63	Inner tegument protein	927	Zhu et al 2005, Bechtel et al 2005	5	540-610: Y.FHIM(+15.99)DILEERHSQDALY.N<br>601-613: L.EERHSQDALYNLL.D<br>134-140: Y.ADQIAGF.K<br>104-129: S.QPPQNTAPAQPPTSDDTLNNC(+57.02)TLLKL.L<br>529-547: Y.DEDIVRSPLFADAFTKSHL.L
ORF64	Large tegument protein deneddylase	2635	Zhu et al 2005	12	893-900: I.TDPNGAHF.H<br>46-58: E.TPLVDRASLDDVL.E<br>779-787: L.QSAATAEHH.L<br>571-585: Y.STAAPSKCTHVLQFF.I<br>892-900: N.ITDPNGAHF.H<br>146-155: A.GAIVVK(+57.02)DK(+42.01)TY.Y<br>1178-1192: H.GEQAWKKIQQAFKDF.N<br>1541-1551: L.S(+75.98)PLRVKGGKAA.V<br>681-706: T.ARLKPNFNIVC(+57.02)ARQDAQTIQDGVGLL.R<br>2397-2410: L.ETKTTPPSTPPHAI.D<br>1566-1574: L.VQEAEQAGL.L<br>90-104: L.RTDDWATKIFQSPEF.Y
ORF65	Small capsomere-interacting protein (SCP)	170	Zhu et al 2005	3	5-24: KVRDPVIQERLDHDYAHHPL<br>78-100: RDQKPRERADRVSAASAYDA GTF<br>148-170: SSTTETAAPAVADARKPPSG KKK
ORF68	Packaging protein UL32 homolog	545	Zhu et al 2005	4	40-57: TQIHQSLQPSSPCRVCQL<br>174-224: KTSWPRTDKEEATGPTPCCQ ITDTTTAPASGIPELARATF CGASRPTKPSL<br>183-195: K.EEATGPTPC(+57.02)C(+57.02)QIT.D<br>355-367: IRGCTPQEIHKHL
ORF75	ORF75	1296	Zhu et al 2005, Bechtel et al 2005	6	124-135: L.QEWARVEVGRHL.V<br>168-192: L.EVPEGPQPVARPHIEDDVIM(+15.99)QAVMI.S<br>180-201: P.HIEDDVIM(+15.99)QAVMISLGADLLPL.A<br>666-690: A.WRGQAMAMGEQAYKMATNVSTGATY.A<br>1011-1024: F.SC(+57.02)PTSPRRVAALVL.P<br>1122-1133: L.GVVGRSESSPYT.Y
K8.1	gp35/37	228	Zhu et al 2005	4	70-121: NGTRLAAGSPSSEYPNVSVS VEDTSASGSGEDAIDESGSG EEERPVTSHVTF<br>123-132: TQSVQATTEL<br>145-151: Y.S(-18.01)SGEPSR.T<br>145-197: SSGEPSRTTRIRVSPVAENG RNSGASNRVPFSATTTTTRG RDAHYNAEIRTHL

Table S5: Hits reported previously by other labs through Mass-Spectrometry and detected in our analysis as well using Chymotrypsin as digesting enzyme

Name	Amino Acid Size	Protein detected	Reported previously by other methods	Number of unique peptide hits	Sequences of Correspondence detected	-10lgP
ORF18	257	Protein UL79 homolog	Gong et al 2014	4	51-81: REATANAGTYDEVVLGRKVP AEVW<br>100-108: L.C(+57.02)EAYRDSLW.M<br>205-226: VWASETGYPGPVEAVCRDIR SM<br>233-248: AVSGYLPAPSEAQLAY	25<br>28.53<br>26.66<br>26.95
ORF32	454	Capsid vertex component 1 (CVC-1)	Dai et al 2014	4	48-79: SRSPGSSRRLVVCGKRVLPG EENQLASSPSGL<br>159-165: R.ATPDPAL.T<br>204-237: DPPVSQKGPARTRHRPPPVR LSFNPVNADVPATW<br>313-334: RQRVPVAIPRDAEIADAVKS HF	25<br>29.43<br>25.01<br>25
ORF38	61	Cytoplasmic envelopment protein 3 (CEP-3)	Wu et al 2016	3	5-28: LSICKRPSQPVDVDGEPLDV VVDY<br>11-28: R.PSQPVDVDGEPLDVVVDY.D<br>41-61: EQSQSPYPALKKKKKNKEAI Y	26<br>45.19<br>25
ORF42	278	Cytoplasmic envelopment protein 1 (CEP-1)	Butnaru et al 2019	3	16-51: STHAPKTRESEEACPVYPHP VVPRLVLEVHRKNNAL<br>133-147: L.ENC(+57.02)RDMSPTFLRSII.C<br>222-243: KLESSGENEDDKQTCADPVN IF	26<br>25.18<br>25
ORF43	605	Portal Protein	DD Dünn-Kittenplon et al, 2019	5	156-163: L.VNSIQEQL.M<br>383-406: L.EKERELC(+57.02)M(+15.99)KRLKC(+57.02)IETQLSHQQPG.D<br>4-19: R.M(+15.99)NPGLGSSISVHPSEL.S<br>383-416:<br>L.EKERELC(+57.02)M(+15.99)KRLKC(+57.02)IETQLSHQQPGDAKGPGSVNL.L<br>429-442: L.QDPSLQLTSSHIPS.G	35.01<br><br>25.49<br>25.53<br>27.14<br>25.97
ORF48	402	ORF48	Sander,G. et al .2007	3	214-221: F.IEHITSDV.R<br>214-223: F.IEHITSDVRR.L<br>260-276: R.KPWKELSVSRINVEARL.L	26.82<br>25.08<br>28.26
ORF56	843	DNA primase	Davis et al 2015	4	247-254: L.ETEAIDRT.I<br>726-735: E.TLSGRSIEDW.L<br>353-360: L.VGERC(+57.02)VYW.C<br>40-46: F.FSVLHDL.F	40.18<br>29.4<br>25.88<br>25.95
ORF K2	204	Viral Interleukin 6 Homolog	Katano,H. et al.2000	7	176-183: Y.VLSAMEKF.A<br>45-55: W.VIDEC(+57.02)FRDLC(+57.02)Y.R<br>178-185: L.S(+154.00)AMEKFAG.Q<br>184-203: F.AGQ(+.98)AVRVLN(+.98)SIPDVTPDVHD.K<br>192-199: L.NSIPDVTP.D<br>8-15:W.SILLVGSL.L<br>17-29: L.V(+42.01)SGTRGK(+42.01)LPDAPE.F	86.52<br>36.29<br>34.9<br>29.75<br>27.62<br>26.81<br>26

Table S6: Hits reported previously by other labs through Mass-Spectrometry and detected in our analysis as well using Chymotrypsin as digesting enzyme

Name	Amino Acid Size	Protein detected	Number of unique peptide hits	Sequences of Correspondence detected	-10lgP
ORF9	1012	DPOL	4	154-164: Y.FYTLAPQGVNL.T<br>323-336: L.HTVGNDKPYTRMLL.G<br>933-940: P.QIHDRIPY.V	34.08<br>30.27<br>27.7

				989-999: F.QNNTSATVAIL.Y	31.12
ORF23	404	ORF23	4	103-124: HSKPVVRGHEFEDTQILPEC RL<br>169-190: DPESPLPRHPITPRAGQTRS IL<br>193-215: H.NIAQVC(+57.02)ERDIVSLNTDNEAASMF.Y<br>325-338: REPIRQPPDCSKVL	25.01<br>26.97<br>27.23<br>25.31
ORF35	150	ORF35	3	2-15: DSTNSKREFIKSAL<br>83-97: RQPRDLPRVADIDAL<br>116-137: EENGEETPTHSSSEIKDTIV RW	25.62<br>28.53<br>25.93
ORF58	357	ORF58	4	65-74: L.C(+57.02)PAAQKQLDR.R<br>116-130: L.LPYPLATATAVYQAP.P<br>215-222: Y.C(+57.02)RREVVVF.V<br>350-357: L.SVINKVVG	27.92<br>29.48<br>25.43<br>25
ORF72	257	viral cyclin homolog	4	1-11: MATANNPPSGL<br>85-93: IKQVSKEHF<br>185-204: VDPKTGSLPASIISAAGCAL<br>206-224: VPANVIPQDTHSGGVVPQL	30<br>26.23<br>25.9<br>25
ORF K3	333	E3 ubiquitin-protein ligase MIR1	3	76-91: L.TYQEGLELIVFIFIMT.L<br>139-152: L.GAFFHM(+15.99)MRHVGRAY.A<br>279-292: Y.VGAQSGDGAYSVSC(+57.02).H	25.04<br>27.54<br>25.13
vIRF-1	449	Viral IRF like protein 1	4	23-73: SQGSPGTSGSGAPCDEPSRS ESPGEGPSGTGGSAAAGDIT RQAVVAAITEW<br>175-189: L.QEIGKGISQDGHHFL.V<br>334-340: L.TSSC(+57.02)NGI.F<br>385-399: ANSPLPAPSHVTCPL	25.03<br>26.47<br>25.44<br>25
vIRF-3	566	Viral IRF like protein 3	3	79-96: GKICTTSRRLRRLPGEEY<br>128-136: L.HSGSSLWEI.L<br>278-293: L.GIPEDVIATSQPGGDT.D	25<br>26.08<br>25.01
vIRF-4	911	Viral IRF like protein 4	5	141-177: L.LSPPMQTTATRRSDTREQSYEEAGAAAPAPPKAPSGL.R<br>492-501: W.SSGGAPNQGL.S<br>672-679: W.EEGLGNIL.S<br>555-572: L.EVEIIDLTGDSDTPST.T<br>871-879: L.SVNPVTC(+57.02)GT.V	26.12<br>30.02<br>29.65<br>25.01<br>25

Table S7: Hits detected in our study using Chymotrypsin as digesting enzyme, not found by Trypsin

Name	Amino Acid Size	Protein detected	Number of unique peptide hits	Sequences of Correspondence detected	-10lgP
ORF44	788	DNA Replication Helicase	5	12-19: D.EPSPGFIL.N<br>55-64: F.DPLEDEGPFL.P<br>225-241:<br>W.LDTPLYRNGAVPC(+57.02)IVC(+57.02)V.G<br>511-520: H.GVKQGHEEFL.R<br>616-622: F.VSTSPGL.H	30.76<br>30.37<br>26.11<br>35.07<br>27.95

Table S8: Example of de novo hits via de novo sequencing feature of PEAKS DB with cut-off ALC% at 75

Sequence	aa Length	ALC%	aa Scoring	PTMs
EC(+57.02)C(+57.02)HGDLLEC(+57.02)ADDRWRR	17	93	98 98 98 97 86 94 95 98 98 97 96 94 94 91 91 82 86	Carbamidomethylation
APSAEVEMTAYVLLAHVTAQPAPGMVGHSK	30	93	98 97 96 96 98 99 100 100 99 99 99 99 100 100 99 99 99 98 97 96 96 96 94 83 89 89 59 60 84 97	N/A
LKEC(+57.02)C(+57.02)DKPLLEK	12	92	97 96 97 99 98 97 96 94 95 78 74 81	Carbamidomethylation
GDSGGPLLLHK	11	88	87 97 97 94 93 97 99 99 85 71 57	N/A
VPLPVSVSVEF	11	87	98 96 99 91 85 73 92 86 73 78 90	N/A

HTM(+15.99)SGVASVESSSGSAVGSPNR	22	85	95 94 89 89 49 71 92 95 97 99 98 99 99 90 96 95 90 71 87 46 54 82	N/A
C(+57.02)VALLTLTY	9	85	96 96 98 99 99 90 73 55 63	Carbamidomethylation
SPQELLC(+57.02)GASLLSDR	15	85	97 97 95 97 99 99 99 88 93 94 93 88 35 41 62	Carbamidomethylation
LLLYALLPDGEVVGDWK	17	84	87 88 79 93 93 97 99 98 97 89 97 97 94 63 78 39 48	N/A
ELNDLLSQLGLRKLF	15	83	95 96 95 99 100 100 98 93 94 30 60 90 89 60 54	N/A
ASFSVLGDLLGSAM(+15.99)R	15	80	68 73 85 94 96 99 96 97 98 96 68 73 66 40 59	N/A
LTESALSPL	9	77	95 67 62 88 54 71 98 90 73	N/A
FLLPQLVK	8	76	92 97 97 86 73 41 41 80	N/A
LFFTHADLGM(+15.99)LEAEQL	16	75	95 91 67 68 94 94 94 94 82 94 95 50 21 21 43 93	Oxidation (M)
RTSTPLPVSVSVEFAVAATDC(+57.02)ALK	24	75	51 55 45 89 93 96 59 55 56 90 91 88 92 96 92 91 92 91 80 75 60 22 48 86	Carbamidomethylation
